# Ciguatera Fish Poisoning and Climate Change: Analysis of National Poison Center Data in the United States, 2001–2011

**DOI:** 10.1289/ehp.1307196

**Published:** 2014-03-11

**Authors:** Daniel B. Gingold, Matthew J. Strickland, Jeremy J. Hess

**Affiliations:** 1 Department of Epidemiology, and; 2 Department of Environmental Health, Rollins School of Public Health, Emory University, Atlanta, Georgia, USA; 3 Department of Emergency Medicine, School of Medicine, Emory University, Atlanta, Georgia, USA

## Abstract

Background: Warm sea surface temperatures (SSTs) are positively related to incidence of ciguatera fish poisoning (CFP). Increased severe storm frequency may create more habitat for ciguatoxic organisms. Although climate change could expand the endemic range of CFP, the relationship between CFP incidence and specific environmental conditions is unknown.

Objectives: We estimated associations between monthly CFP incidence in the contiguous United States and SST and storm frequency in the Caribbean basin.

Methods: We obtained information on 1,102 CFP-related calls to U.S. poison control centers during 2001–2011 from the National Poison Data System. We performed a time-series analysis using Poisson regression to relate monthly CFP call incidence to SST and tropical storms. We investigated associations across a range of plausible lag structures.

Results: Results showed associations between monthly CFP calls and both warmer SSTs and increased tropical storm frequency. The SST variable with the strongest association linked current monthly CFP calls to the peak August SST of the previous year. The lag period with the strongest association for storms was 18 months. If climate change increases SST in the Caribbean 2.5–3.5°C over the coming century as projected, this model implies that CFP incidence in the United States is likely to increase 200–400%.

Conclusions: Using CFP calls as a marker of CFP incidence, these results clarify associations between climate variability and CFP incidence and suggest that, all other things equal, climate change could increase the burden of CFP. These findings have implications for disease prediction, surveillance, and public health preparedness for climate change.

Citation: Gingold DB, Strickland MJ, Hess JJ. 2014. Ciguatera fish poisoning and climate change: analysis of National Poison Center data in the United States, 2001–2011. Environ Health Perspect 122:580–586; http://dx.doi.org/10.1289/ehp.1307196

## Introduction

Ciguatera fish poisoning (CFP) is the most common nonbacterial illness associated with fish consumption, affecting 50,000–200,000 people annually (Dickey and Plakas 2010). Ciguatera toxin is produced by benthic dinoflagellate plankton in the genus *Gambierdiscus*, which live on dead coral surfaces and bottom-dwelling algae. Toxin accumulates in tissues of fish that eat the algae and bioaccumulates up the food chain (Tester et al. 2010). Humans eating contaminated fish are susceptible to the toxidrome caused by the ciguatera toxin, which includes gastrointestinal upset followed by neurologic symptoms including paresthesias and hot–cold reversal (Friedman et al. 2008).

CFP is a significant public health problem in endemic areas, including the Caribbean and Pacific Islands. CFP prevalence in these areas is affected by El Niño Southern Oscillation (ENSO/El Niño) and warm sea surface temperature (SST) conditions (Hales et al. 1999; Llewellyn 2010; Tester et al. 2010). CFP is most prevalent in tropical regions of warm, stable SSTs that remain above 24°C, and laboratory studies have shown that water temperatures of 29°C are optimal for *Gambierdiscus* growth (Hales et al. 1999; Tester et al. 2010). Climate change is projected to expand the range of suitable habitat for the organisms that cause ciguatera by expanding the range of warm SSTs and bleaching coral reefs (Moore et al. 2008). The increase in storm frequency also damages reefs and increases nitrate-rich soil runoff, which may increase the growth of marine algae and ciguatera toxin-producing organisms (Swift and Swift 1993).

We hypothesized that CFP incidence in the United States is associated with warm Caribbean SST and high severe storm frequency. If true, projections of increased SST and storm frequency resulting from climate change suggest CFP incidence in the United States may increase. In addition, this study provided an opportunity to investigate the epidemiology of CFP in the United States and to evaluate the suitability of the National Poison Data System (NPDS) for use in CFP surveillance.

## Methods

We used data from multiple sources to perform a time-series analysis exploring plausible associations between SST, storm frequency, and CFP incidence. First, we produced descriptive statistics on timing and location of CFP calls. We also evaluated single variable associations between candidate variables (which were created with a range of lag times and parameterization techniques) and CFP calls to identify the lag time and weather variable parameterization technique most associated with CFP calls. Using these results, we developed a final model using stepwise selection, including variables most strongly correlating with CFP calls and most consistent with known ecological mechanisms linking weather and CFP. Finally, we applied rate ratios from this model to projected climate scenarios to hypothesize the magnitude of the effect of weather on ciguatera cases in the United States. Additional details regarding data sources, treatment, and analysis are presented below. We also present results from the single-variable analysis to guide the reader through our selection of variables for the final model and to inform future investigation into optimizing weather variable parameterization for association with CFP.

*Ciguatera cases data*. Calls to U.S. poison control centers with the substance code “Ciguatera Fish Poisoning” affecting humans during 2001–2011 were obtained from the NPDS run by the American Association of Poison Control Centers (http://www.aapcc.org). We obtained data for all calls from the lower 48 states, the District of Columbia (DC), Puerto Rico (PR), and the U.S. Virgin Islands (USVI) (*n* = 1,124). Poison center calls are coded with the ciguatera substance code if information about ciguatera toxin is discussed. From these, we selected the subset of calls where ciguatera fish poisoning was the sole substance code listed (*n* = 1,102). Calls coded with “confirmed nonexposure” were excluded from analysis. Day, month, and year were available for all recorded calls. Location was coded by the state or country from which the call originated. Data regarding where the exposure occurred, type/origin of fish consumed, and other narrative notes were unavailable for analysis. Calls were assigned regions and division locations according to the U.S. Census Bureau regions.

Key variables in the descriptive analysis included date, state, and ZIP code of call; caller site (e.g., home, work); exposure site (e.g., home, work); age in years [134 missing values (however, some of these were coded as “unknown adult,” “child,” “teen,” for example, and were used in coding the age categorical variables)]; sex of patient; outcome; reason/route; clinical symptoms reported; clinical effect; and therapy. Outcomes were determined by the poison information specialist coding the call at the conclusion of the case, based on available information on the severity and duration of symptoms and the need for treatment as a result of the exposure. “Major effects” were symptoms that are life threatening or result in significant residual disability or disfigurement. “Moderate” effects were less pronounced symptoms with no residual effects but that typically required treatment. “Mild” effects had short duration and were minimally bothersome.

*SST data*. Monthly SST data from years 1999–2011 contained in the Reynolds/National Oceanic and Atmospheric Administration (NOAA) (OI.v2) SST Data Set were downloaded from the IRI/LDEO Climate Data Library at Columbia University (International Research Institute for Climate and Society 2012). Weekly SST values are created on a 1° × 1° spatial grid from ship, buoy, and satellite measurements as well as SST simulated by sea-ice cover. (Reynolds and Smith 1995; Reynolds et al. 2002). Monthly values are created by linear interpolation of weekly data to daily fields, and averaging daily values in a 1° global spatial grid. Land-masking was used to remove values for SST that were over land. Monthly maximum and minimum SSTs in the Caribbean were found in bounds of 7°N–35°N latitude and 97°W–40°W longitude (exclusive of the Pacific Ocean included in this area). Maximum and minimum monthly SST values along 34.5°N and 24.5°N were found by restricting data to a 1° band around these parallels. Monthly maximum and minimum latitudes for the 25°C and 29°C contours were found by limiting the monthly data to all measurements within 0.25°C of the desired contour and finding the maximum or minimum latitude value for each month. Yearly maximums (minimums) were found by taking the largest (smallest) monthly value for that year. To create the peak August temperature variable, we assigned the maximum temperature value in August over the Caribbean region of a given year to all months of the same calendar year; the same was done for the nadir March temperature variable using the minimum temperature value in March.

*Caribbean SST anomaly index data*. Monthly Caribbean SST index values from years 1999–2011 were obtained online from the Earth System Research Laboratory (NOAA 2013). SST forecasts, based on NOAA Extended Reconstructed Sea Surface Temperatures (version 3b, employing linear inverse modeling) were used to calculate anomalies relative to 1981–2010 climatology (Penland and Matrosova 1998). These anomalies were averaged over the Caribbean (bounded by 26°N, 80°W, and the eastern coast of Central America) to create a monthly SST index value, identified by NOAA as the “CAR index,” representing the deviation of current SST in the entire Caribbean region from historical averages.

*Severe tropical storms data*. Data for severe tropical storms (tropical depressions, tropical storms, and hurricanes) for years 1999–2011 are available from Unisys Weather (2012). Storms were assigned to the months in which they began. Total storm days for a month are the sum of the durations of all storms that began in that month. Severe storms are indicated by a category ≥ 3 hurricane, according to the Saffir-Simpson scale. Data for accumulated cyclone energy, a measure of the strength and duration of storms over a monthly period, is available from NOAA (Maue 2011; Policlimate 2012).

*Fishing yields data*. Caribbean fishing yields during years 2001–2010 are available from the Food and Agriculture Organization of the United Nations (2012). The online query tool for global capture production provided yearly totals for fishing capture of marine and diadromous fishes in marine areas for the Western Central Atlantic Ocean (bounded by 35°N, 40°W, and the eastern coasts of North, Central, and South America). These data are in metric tons and are reported yearly. Fishing yields for 2011 were estimated using data from the previous decade based on a linear regression model.

*Lagged variables*. We created lagged variables for weather explanatory variables for 3, 6, 12, 18, and 24 month lags. A 3-month lag assigns the value of the original variable in January to the following April. Lagged variables for peak and nadir variables do not have a constant lag period. For example, the lag periods for the August maximum SST variable of the previous calendar year are between 5 and 16 months: January values refer to the most recent August 5 months earlier, but December values refer to the August 16 months earlier.

*Analysis methods*. We used Poisson regression to estimate associations between monthly CFP incidence and SST and storm frequency, using regional annual captured fish production yields as the offset (to compensate for the effect of changing fishing yields). Candidate explanatory variables included severe storm totals (by category), total storm days, monthly SST anomaly, each month’s maximum and minimum SST in the Caribbean region and along the 34.5°N and 24.5°N parallels, and the maximum and minimum latitudes achieved each month by the 25°C and 29°C SST contours, as well as variables created to reflect the August maximum and March minimum temperatures for each year. We evaluated pairwise correlations between candidate variables using Pearson correlation coefficients (*r*).

We evaluated the association between CFP calls and explanatory storm and SST variables using 0-, 3-, 6-, 12-, 18-, and 24-month time lag windows, controlling for month with dummy variables and using the yearly regional fishing yields as an offset. Direction and magnitude of the regression coefficients were examined graphically to identify the lag period for each variable that achieved the highest statistical significance by the Wald chi-square statistic. SEs were scaled by the Pearson’s chi-square statistic in order to inflate the SEs of the model’s β coefficients to account for an overdispersion of observed data relative to the Poisson assumption of variance being equal to the mean. We selected variables for inclusion in the multivariate model based on the individual variable analysis. The multivariate model retained the monthly dummy variables and offset. We used rate ratios from the multivariate model to estimate the expected number of ciguatera calls due to a 10% or 25% increase in storm frequency (Henderson-Sellers et al. 1998; Oouchi et al. 2006) and 2.5°C or 3.5°C increase in SST (Christensen et al. 2007; Moore et al. 2008; Sheppard and Rioja-Nieto 2005). All analysis was performed using SAS, version 9.3 (SAS Institute Inc., Cary, NC).

## Results

There were 1,102 calls exclusively coded as CFP calls and made from the lower 48 states, PR, DC, or USVI. Table 1 presents descriptive statistics for these calls. The South census region, which includes Florida and other Gulf and Atlantic coast states, had the largest proportion of calls (62.2%). A total of 412 calls (37.4%) resulted in moderate or major clinical effect (including death). The most common symptom reported was diarrhea (39.3%), followed by vomiting (32.1%) and numbness (22.3%) (data not shown). Although volatile, yearly CFP totals demonstrated a moderate overall increase in CFP calls across the decade of available data (Table 1; see also Supplemental Material, Figures S1 and S2).

**Table 1:** Descriptive statistics of ciguatera-related calls to poison centers, 2001–2011 [*n* (%) except where noted].

Variable	Lower 48 states, PR, USVI true cases (*n* = 1,102)
Sex (male)^*a*^	515 (48.1)
Age (years)^*a*^
Median [years (IQR)]	40 (28–51)
< 18	91 (10.0)
20–29	150 (15.3)
30–39	211 (21.5)
40–49	234 (23.8)
50–59	163 (16.6)
60–69	87 (8.9)
≥ 70	29 (2.9)
Region^*a*^
Midwest	79 (7.2)
Northeast	174 (15.8)
Other (PR, USVI, territories, overseas)	12 (1.1)
South	685 (62.2)
West	152 (13.8)
Outcome
Death	1 (0.1)
Major effect	26 (2.4)
Minor effect	237 (21.5)
Moderate effect	385 (34.9)
No effect	29 (2.6)
Not followed, nontoxic exposure	11 (1.0)
Not followed, minor effect	232 (21.1)
Unable to follow, potentially toxic	139 (12.6)
Unrelated effect, exposure probably not responsible	42 (3.8)
Year of call^*a*^
2001	70 (6.4)
2002	92 (8.4)
2003	86 (7.8)
2004	72 (6.5)
2005	61 (5.5)
2006	95 (8.6)
2007	116 (10.5)
2008	123 (11.2)
2009	127 (11.5)
2010	137 (12.4)
2011	123 (11.2)
^***a***^No. of missing values: sex (32), age (223), region (5), year of call (139).	

Table 2 shows descriptive statistics for candidate monthly variables for regression; for conciseness these results are presented for alternating months. There was a clear seasonal pattern of CFP calls, with more calls being made during the summer months. As expected, there was a seasonal pattern to both storms and SST data: During summer months, water temperatures were warmer and extended farther northward, and storms were more frequent. Pairwise correlations among these variables are shown in Supplemental Material, Table S1. Positive correlations were present for all of the variables except the minimum latitude of the 29° contour. The August maximum regional SST was positively correlated with the August CAR index (*r* = 0.88, *p* < 0.001) and mean yearly CAR index (*r* = 0.66, *p* = 0.014) and less so with yearly storms (*r* = 0.41, *p* = 0.16). Yearly accumulated cyclone energy was correlated with yearly total storms (*r* = 0.75, *p* = 0.003) (see Supplemental Material, Table S1). Trends of yearly calls, storms, and maximum August SST in the Caribbean are shown in Supplemental Material, Figures S1 and S2. Although yearly CFP call totals generally increased over the decade, there was less of an obvious pattern to peak temperatures and storm frequency, which peaked in 2005.

**Table 2:** Descriptive statistics for regression analysis variables, 2001–2011.

Variable	February	April	June	August	October	December	All year
Ciguatera cases
Ciguatera cases in lower 48 states, DC, PR, USVI coded exclusively as ciguatera substance
Mean ± SD	6.6 ± 3.4	7.3 ± 4.4	11.0 ± 3.8	15.3 ± 9.3	6.4 ± 3.1	4.9 ± 3.3	100.2 ± 26.3
Range	4–16	3–17	6–18	3–28	2–12	1–12	61–137
SST
CAR Index ± 1981–2010 climatology
Mean ± SD	0.1 ± 0.17	0.16 ± 0.15	0.15 ± 0.13	0.16 ± 0.11	0.16 ± 0.12	0.1 ± 0.14	0.1 ± 0.1
Range	–0.13–0.36	–0.03–0.41	–0.06–0.4	–0.06–0.35	–0.05–0.41	–0.14–0.29	–0.2–0.4
Maximum SST in Caribbean
Mean ± SD	29.6 ± 0.5	30.3 ± 0.3	30.1 ± 0.3	30.6 ± 0.3	30 ± 0.2	29.3 ± 0.3	30 ± 0.5
Range	29–30.3	29.7–30.7	29.6–30.6	30–31.2	29.7–30.3	29–29.9	28.7–31.2
Minimum SST in Caribbean
Mean ± SD	29.6 ± 0.5	30.3 ± 0.3	30.1 ± 0.3	30.6 ± 0.3	30 ± 0.2	29.3 ± 0.3	20.7 ± 3.8
Range	13.6–17.4	17.7–18.7	21.1–22.8	25.8–26.6	23.1–24.2	17.5–19.7	13.6–26.7
Maximum latitude of 25°C SST
Mean ± SD	25.8 ± 0.8	27.6 ± 1.9	37 ± 1.3	41 ± 0.5	38.4 ± 0.9	28.9 ± 2.1	32.8 ± 6
Range	24.5–26.5	25.5–32.5	34.5–38.5	40.5–41.5	36.5–39.5	26.5–32.5	24.5–41.5
Minimum latitude of 25°C SST
Mean ± SD	14.5 ± 2.4	14.9 ± 2.7	23 ± 5.8	38.4 ± 0.9	30.8 ± 1.3	19.7 ± 1.6	23.7 ± 8.9
Range	12.5–19.5	12.5–20.5	13.5–28.5	36.5–39.5	29.5–32.5	18.5–23.5	12.5–39.5
Maximum latitude of 29°C SST
Mean ± SD	13.5 ± 0	15 ± 0.7	27.4 ± 2.5	31.6 ± 2.2	24.3 ± 1.5	13.3 ± 0.4	21.4 ± 7.3
Range	13.5–13.5	13.5–15.5	23.5–29.5	28.5–34.5	21.5–26.5	12.5–13.5	12.5–34.5
Minimum latitude of 29°C SST
Mean ± SD	13.5 ± 0	10.5 ± 0	10.5 ± 0	10.5 ± 0	10.5 ± 0	12.8 ± 0.9	11.1 ± 1.1
Range	13.5–13.5	10.5–10.5	10.5–10.5	10.5–10.5	10.5–10.5	10.5–13.5	10.5–13.5
Maximum SST at 24.5°N latitude
Mean ± SD	25.4 ± 0.4	26.3 ± 0.4	29 ± 0.4	30.3 ± 0.2	28.8 ± 0.3	26.4 ± 0.5	27.7 ± 1.8
Range	24.9–26.4	25.8–26.9	28.7–30	29.9–30.6	28.3–29.4	25.6–27.1	24.8–30.6
Maximum SST at 34.5°N latitude
Mean ± SD	20.6 ± 0.7	21.7 ± 0.8	26.3 ± 0.6	28.3 ± 0.5	25.9 ± 0.3	22.5 ± 0.5	24.1 ± 2.9
Range	19.5–22	20.5–23.4	25.5–27.5	27.4–29.1	25.4–26.3	21.5–23.2	19.1–29.1
Minimum SST at 24.5°N latitude
Mean ± SD	21.2 ± 0.6	23.1 ± 0.4	25.4 ± 0.5	26.6 ± 0.5	27 ± 0.3	23.4 ± 0.5	24.4 ± 2.1
Range	20.2–22.1	22.3–23.7	24.1–25.8	25.9–27.4	26.4–27.4	22.7–24	20.1–28.1
Minimum SST at 34.5°N latitude
Mean ± SD	17.8 ± 0.7	18.4 ± 0.4	22.1 ± 0.5	26.5 ± 0.3	23.7 ± 0.3	19.9 ± 0.8	21.4 ± 3.1
Range	16.8–18.7	17.8–18.9	21.1–22.8	26.2–27.2	23.1–24.2	18.2–20.7	16.8–27.2
Severe storms
Total storms							
Mean ± SD	0 ± 0	0.09 ± 0.3	0.73 ± 0.79	4.64 ± 1.91	2.64 ± 2.25	0.27 ± 0.47	17.5 ± 5.8
Range	0–0	0–1	0–2	3–8	0–7	0–1	9–31
Hurricanes							
Mean ± SD	0 ± 0	0 ± 0	0.09 ± 0.3	1.64 ± 1.12	1.36 ± 1.8	0.09 ± 0.3	7.8 ± 3.7
Range	0–0	0–0	0–1	0–4	0–5	0–1	3–16
Tropical depressions							
Mean ± SD	0 ± 0	0 ± 0	0.09 ± 0.3	0.45 ± 0.52	0.27 ± 0.47	0 ± 0	2.1 ± 1.4
Range	0–0	0–0	0–1	0–1	0–1	0–0	0–5
Tropical storms							
Mean ± SD	0 ± 0	0.09 ± 0.3	0.55 ± 0.69	2.55 ± 1.13	0.82 ± 0.87	0.18 ± 0.4	7.6 ± 2.2
Range	0–0	0–1	0–2	1–5	0–2	0–1	4–11
Category ≥ 3 storms							
Mean ± SD	0 ± 0	0 ± 0	0 ± 0	1.09 ± 0.83	0.55 ± 0.82	0 ± 0	3.7 ± 1.7
Range	0–0	0–0	0–0	0–2	0–2	0–0	2–7
Total storm days							
Mean ± SD	0 ± 0	0.91 ± 3.02	5.1 ± 5.5	35.5 ± 15.6	16.9 ± 13.9	2.0 ± 3.5	138 ± 46.7
Range	0–0	0–10	0–15	17–66	0–43	0–9	70–245
Accumulated cyclone energy
Mean ± SD	0 ± 0	0.2 ± 0.8	1.5 ± 1.8	26.9 ± 17.8	17.7 ± 15.4	1.8 ± 4	132.7 ± 66
Range	0–0	0–2.6	0–5.8	3.4–64.7	2.6–50.9	0–13.2	51–250
Values are the mean monthly value from years 2001–2011 ± SD along with range (minimum–maximum) shown for each variable for alternating months.							

**Figure 1:**
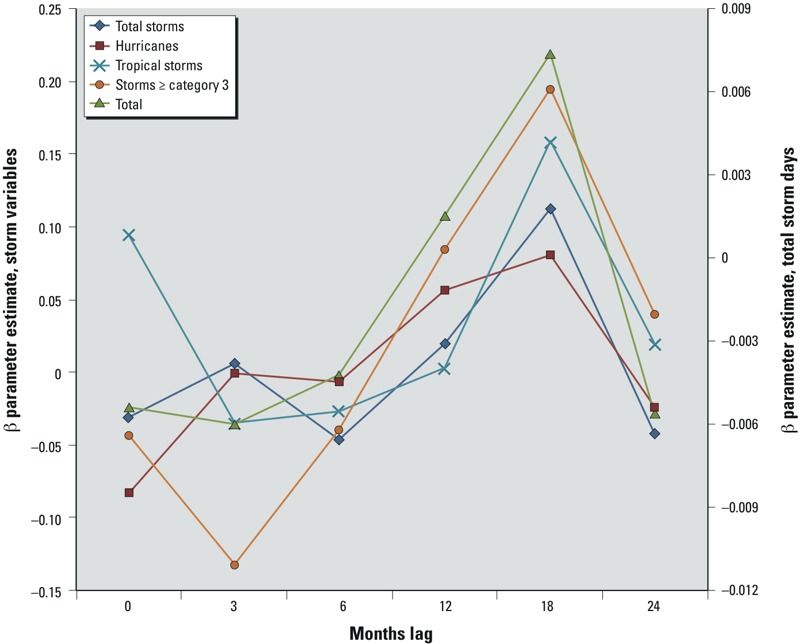
Poisson β estimates by lag time for selected tropical storm variable. The β parameter estimate for single variable Poisson regression (controlling for month) for total storm days is plotted on the right y‑axis. β parameter estimates for all other variables are plotted on the left y‑axis.

Results from the univariate analyses are shown in Supplemental Material, Tables S2 and S3. Parameter estimates for the storm variables by lag time are displayed graphically in Figure 1. The β coefficient estimates were largest at the 18-month lag time, at which point the total storms variable (which had the most observations) was significantly associated with the number of CFP calls (Wald chi-square statistic = 4.22, *p* = 0.04) (see Supplemental Material, Table S2). A similar pattern was less apparent among the SST variables. Whereas 12 months is a relatively consistent inflection point, most of these associations were not statistically significant (see Supplemental Material, Tables S2 and S3). Among peak variables, the peak August SST for the previous calendar year variable was positively associated with the outcome in the individual variable model (*p* = 0.004). There were other peak and nadir variables that were associated with the outcome, although most lost significance when included in a multivariate model.

Candidate variables for the multivariate model were identified by the strength of their association with the outcome in the univariate analysis (see Supplemental Material, Tables S2 and S3). Many variables no longer had significant parameter estimates when included simultaneously in the Poisson model. Significant associations persisted with the total storm variable lagged at 18 months and the peak Caribbean SST from August of the preceding calendar year (variable 5- to 16-month lag); these variables were included as the explanatory variables in the final multivariate Poisson model (see Supplemental Material, Table S4). The rate ratio for an increase of one severe storm per month was 1.11 (95% CI: 1.03, 1.23) and the rate ratio of an increase in SST of 1°C/month was 1.61 (95% CI: 1.17, 2.24) (Table 3). These rate ratios did not change substantially with the inclusion of borderline variables in the multivariate model (see Supplemental Material, Table S4). Restricting ciguatera cases to only those with moderate or major clinical effects resulted in rate ratios of similar values, although confidence intervals were wider (and included the null) because the number of calls was reduced by 65% (see Supplemental Material, Table S5). Limiting fishing yields to fish species commonly found to be ciguatoxic resulted in rate ratios for SST between 1.2 and l.4, consistent with our original model’s results, although the 95% CIs do not include one. Statistical significance varied depending on which fish were used, but most excluded the null. Rate ratios for storms and their statistical significance did not change under this sensitivity analysis (see Supplemental Material, Table S5).

**Table 3:** Rate ratios and excess CFP calls expected for hypothetical scenarios compared with 2001–2011 baseline.

Hypothetical scenario	Rate ratio ± SE (95% CI)	Extra CFP calls (95% CI)
Increase in 1 storm/month	1.11 ± 0.06 (1, 1.23)	11.3 (0.4, 23.4)
Increase in 1°C for 1 month	1.62 ± 0.27 (1.17, 2.24)	61.9 (16.7, 124.5)
Increase in 1°C for 1 month and 1 storm/month	1.8 ± 0.31 (1.28, 2.53)	80.1 (28.3, 153)
Increase in storm frequency of 10%	1.02 ± 0.01 (1, 1.03)	1.6 (0.1, 3.1)
Increase in storm frequency of 25%	1.04 ± 0.02 (1, 1.08)	4 (0.1, 8)
Increase in maximum August SST 2.5°C	3.33 ± 1.39 (1.47, 7.53)	233.3 (47.2, 654.4)
Increase in maximum August SST 3.5°C	5.38 ± 3.14 (1.72, 16.89)	439.3 (71.8, 1592)
Increase in maximum August SST 2.5°C and increase in storm frequency of 10%	3.38 ± 1.41 (1.49, 7.65)	238.5 (49.5, 665.9)
Increase in maximum August SST 3.5°C and increase in storm frequency of 25%	5.6 ± 3.26 (1.79, 17.55)	460.7 (78.7, 1658)
This table applies the final multivariate model to several possible weather scenarios based on current climate prediction models for the Caribbean region.		

As climate change progresses, the effect of increases in SST on storm frequency is uncertain. Table 3 shows several possible scenarios, with predictions in CFP cases provided by the final multivariate model. Percent change in storms is relative to the baseline of our data, years 2001–2011, during which there was an average of 17.5 storms/year (1.46/month) and 100.2 CFP calls/year (8.35/month) (Table 2). For example, if the maximum SST in the Caribbean increases by 2.5°C as projected, and storm frequency increase by 10%, then 238.5 additional calls per year can be expected based on the regression model results (95% CI: 49.5, 665.9) (Table 3). These estimates assume that population, rate of fish consumption, clinical recognition, and other factors affecting CFP calls in the United States remain similar to 2001–2011 levels.

## Discussion

*Summary*. We found that the monthly ciguatera-related calls to poison control centers in the continental United States were associated with both tropical storm frequency and peak SST in the Caribbean basin during the study period. An increase of one storm per month (lagged 18 months) was associated with an increase in CFP calls of 11% (95% CI: 0.4, 23), whereas an increase in 1°C SST during August of the previous calendar year was associated with a 62% (95% CI: 17, 1,242) increase in calls. Because SST and storms are positively correlated, any meteorological change that causes one of these factors to increase will likely cause an increase in the other as well.

Recent increases in Caribbean SST are very likely due to climate change, and projections predict an increase of ≥ 2.5°C during the 21st century (Christensen et al. 2007; Moore et al. 2008; Sheppard and Rioja-Nieto 2005). Increases in storm frequency are less certain, although regional storm frequency has been linked to SST and it is possible that storm frequency will increase in the North Atlantic (and it is very likely that storms will become more intense) (Christensen et al. 2007; Oouchi et al. 2006). Our model suggests that for moderate increases in both SST and storms, the effect of SST would dominate, with the effect of storm increases accounting for a small proportion of the expected increase in CFP calls (Table 3). In these scenarios, our model estimates that 200–400 calls a year could be attributable to climate change, assuming constant population and fish consumption. This represents a large (200–400%) increase in yearly CFP incidence (using poison center calls as a marker for true incidence) relative to 2001–2011 baseline. Although these estimates are uncertain, they imply that the fears of an increase in CFP in the United States may be justified and that this increase may be significant from both a clinical and public policy perspective.

*Comparison of results to literature*. Using the yearly average calls of 100 and U.S. population of 300 million (roughly average for the decade), our data set had a yearly call incidence of 0.003/10,000 residents. This is significantly lower than other reports in the U.S. literature (0.3/10,000 in Hawai’i, 5/10,000 in Dade County, FL) (Azziz-Baumgartner et al. 2012; Friedman et al. 2008). However, using an expected reporting rate of 1%, this may represent a true yearly incidence of 0.3/10,000, which is a reasonable order of magnitude given that the study area included both high and low CFP incidence areas (Lawrence et al. 1980). Incidences of CFP calls in PR and USVI were lower than previously reported survey data likely because residents in CFP endemic areas are familiar with the disease and probably rarely seek assistance from health professionals or poison control centers (Azziz-Baumgartner et al. 2012). Numbers of CFP calls identified in our data are consistent with U.S. incidence estimates in previous literature, as is the seasonal trend of more cases in the summer than winter months (Tosteson et al. 1988).

Commonly associated symptoms in our data set were similar to previous reports (Azziz-Baumgartner et al. 2012; Friedman et al. 2008), although the percentages of observations with each type of symptom were generally lower in our data. This may be because our data has more low-severity or unconfirmed cases, and the reports in the literature are more likely to be confirmed and therefore more likely to have more substantial clinical effects.

The literature provided little guidance on choice of lag structure for the regression variables. For SST lags, previous literature reported ≥ 13 months as appropriate (Parsons et al. 2010). Our 5- to 16-month lag is consistent with previous work as well as the proposed mechanism of the effect of weather on CFP, in which warmer SSTs increase ciguatoxic organism growth that, after bioaccumulation, increases human exposure to ciguatera toxin. The literature had no guidance for storms, but we expected the lag time for the effect of storms (18 months) to be longer than that of SST because the proposed mechanism adds habitat destruction and recolonization by toxic dinoflagellates to the SST timeline. More research must be done to better measure and understand these lag structures.

Notably, 2005 was a peak year for SST and storms in our data, possibly due to an ENSO/El Niño warming event during the last part of 2004 and early 2005 (NOAA 2012). Globally, 2005 was the hottest year since 1880 to date, and it has only been matched by 2010 since then (Hansen et al. 2010; National Aeronautics and Space Administration 2011). There has been some evidence that Pacific CFP is affected by ENSO events; however, calls did not peak in 2005, although the years after 2005 did experience a rise in call frequency (Hales et al. 1999).

*Limitations*. There are several limitations to our data and approach. Our outcomes are not confirmed cases. As mentioned above, poison center calls are coded with the ciguatera substance code if information about ciguatera toxin is discussed. Often there are several codes for similarly presenting illnesses and the call is to discuss this differential diagnosis. Follow-ups to confirm the diagnosis are rare, although ciguatera is always a clinical diagnosis because there are no reliable diagnostic tests or biomarkers for its detection in humans. Apart from a reduction in the severity of symptoms associated with cases identified in this way, it is difficult to determine the impact of this potential bias on the findings.

More importantly, the information about the location of the call is limited to what call center took the call and does not include exposure location or fish origin. It is common for travelers to seek medical attention for symptoms upon return from a trip. Therefore, we chose to correlate calls with Caribbean basin weather instead of making the resolution of these data finer than is warranted by the method of data collection. We assumed broadly that fish causing a ciguatera-associated illness in the continental United States were very likely to have come from the Caribbean and to have been exposed to the ecological and meteorological conditions there. Given that most U.S. cases are from regions where CFP is locally endemic, this is not an unreasonable assumption. We also assumed that although ciguatoxin can survive freezing and other food preservation methods, fish are consumed around the same time (i.e., the same year) they are harvested (Friedman et al. 2008). Lag times are meant to incorporate the ecological lag time between meteorological conditions that increase ciguatoxic dinoflagellate production and the increased number of ciguatoxic fish harvested. We speculated that biases from these limitations and assumptions would result in an underestimate of true effects.

Using poison center calls also likely resulted in an underestimate of effect because CFP has been shown to be widely underreported due to nonspecific symptoms, frequent low acuity, and low awareness among U.S. medical providers (Lawrence et al. 1980). We viewed calls to poison centers as a proxy for true incidence and although we expected trends in both calls and true incidence to be similar, it is likely that the number of calls per year grossly underestimated the true incidence. It is possible that changes in clinical awareness of the disease that occurred over the decade may have resulted in detection bias, although it is not clear why this would be associated with weather trends (which did not necessarily increase uniformly across the decade).

We did not control for changes in tourism to endemic areas, and, although this is not as likely to be correlated with annual variations in spring as summer SST, it could conceivably be related to storm frequency. However, it is difficult to hypothesize a relationship that would link tourism with the lag structures we observed. Similarly, we did not control for changes in fishing or eating practices that could confound the relationship, such as seasonal fishing closures or prohibition of sale of high-risk fish species from ciguatoxic areas. It is likely that the effect of these behaviors would be very local, not related to regional weather, and would underestimate the effects of weather on CFP incidence. Changes in SST, storms, or decreased fishing yields might lead to changes in types and ages of fish captured or cause fishermen to seek out fish in alternate areas previously avoided due to concerns for CFP. This may or may not alter the likelihood of harvesting ciguatoxic fish. We included fish production in our model in an attempt to control for any systemic regional effect of these behaviors. An increase in the awareness of medical providers regarding ciguatera poisoning during the study period is another potential uncontrolled-for confounder.

With only 10 years of ciguatera call data, we could not estimate the effect of climate change (i.e., a warmer, stormier climate) on ciguatera incidence. Instead, we can only examine the effect of climate variability: that is, correlations between warmer or stormier years and call incidence. Such a relationship supports the theory that as the regional climate warms and storm frequency increases as projected by climate models, all other things being equal, the range of ecological suitability for ciguatoxic organisms and the burden of CFP are likely to increase in the United States.

Selecting variables for the final multivariate model was challenging. Examining a large number of candidate variables to represent SST may have increased the possibility of type 1 error. Ultimately the variable selected was a relatively intuitive and broad regional measure of SST and had a lag structure similar to that presented in the literature. We have higher confidence in the validity of the storm variable we selected because the pattern between lag months and β parameter significance was as we anticipated *a priori*. Even though the magnitude of effect for our SST variable is larger than that for the storms, and therefore is the main driver of projected CFP increase, we must acknowledge greater uncertainty in the validity of the structure and measurement of the SST variable compared to the storm variable.

Finally, our future projections of CFP disease burdens may be overestimates: They do not take into account changes in technology, detection, and clinical and public education and awareness that may affect both the detection of the disease and its incidence. If CFP does indeed increase in the United States as projected, implementation of clinical and public health measures to reduce transmission of the toxin may blunt the expected rise in cases.

*Public health implications*. Increased incidence of CFP in the United States would have numerous public health impacts. Burden of disease would increase, resulting in increased utilization of health care resources, particularly poison control centers and emergency departments. Education targeting emergency providers and toxicologists may help address these concerns. Further, areas that have not experienced high levels of CFP and therefore have low awareness of the condition might begin to see the disease more frequently. Adaptation measures such as education of health professionals (Hess et al. 2009) and the public, as well as enhanced surveillance (Frumkin et al. 2008), may mitigate these risks. Preventive strategies, such as regulating fishing industry catches and imports, may prove necessary, although the development of a method for identifying ciguatoxic fish will be essential in developing more effective surveillance, monitoring, and prevention strategies. Further characterization of the temporal and spatial relationships between storms, SST, and CFP may enable the development of a weather-based early warning system for CFP outbreaks that could better target prevention strategies.

## Conclusions

This hypothesis-generating study substantiates concern that CFP could increase in the United States due to climate change, despite limitations discussed above. More specific data on outcomes, exposures, and ecological factors are all needed to investigate these findings further. The NPDS data were sufficient for this study but likely do not accurately represent the epidemiology of the disease in the United States. Data on cases with confirmed disease (or emergency department reports from state and federal sources), perhaps initially from high-incidence areas, may be better suited to identifying weather-related patterns. Additional ecological data regarding changes in the distribution of suitable habitat for ciguatoxic dinoflagellates (due to SST warming, coral bleaching events, man-made structures, and storm damage) and their overlap with U.S. fishing areas would help clarify exposure pathways. Identifying toxic fish and their origin would also be very useful in identifying ciguatoxic areas, allowing more specific analysis for what weather and climate parameters play a role in CFP. Finally, more work must be done to identify the proper lag time between weather and SST disturbance and CFP incidence and to determine if the length of this lag period is dependent on severity or type of weather disruption. As more data are gathered on CFP and its ecology, associations between longer climate variability and CFP incidence can be investigated. Despite these concerns, the results of this study are consistent with others signaling that CFP may become more prevalent as the climate continues to change, and appropriate investment in public health preparedness is prudent.

## Supplemental Material

(401 KB) PDF
