# Supplementary material for: Ciguatera Fish Poisoning and Climate Change: Analysis of National Poison Center Data in the United States, 2001–2011
Source: Environ Health Perspect. 2014 Mar 11;122(6):580–6. doi: 10.1289/ehp.1307196 (PMC4050511; doi:10.1289/ehp.1307196)
Supplement: (401 KB) PDF [file ehp.1307196.s001.pdf]

Supplemental Material

Ciguatera Fish Poisoning and Climate Change: Analysis of National Poison Center Data in the United States, 2001−2011

Daniel B. Gingold, Matthew J. Strickland, and Jeremy J. Hess

Table of Contents **Page**

Table S1. Pairwise correlation, Pearson coefficients **2**

Table S2. Individual variable regression results, Wald χ^2 statistic **3**

Table S3. Individual variable regression results, β estimate **4**

Table S4. Sensitivity of beta estimates to multivariate model selection **5**

Table S5. Sensitivity of rate ratios to outcome and offset restrictions **6**

Figure S1. Total storms and CFP calls by year **7**

Figure S2. August max SST and CFP calls by year **8**

Table S1. Pairwise correlation, Pearson coefficients.

	**Total storms**	**Hurricanes**	**Tropical Depressions**	**Tropical Storms**	**Storm Category > 2**	**Total storm days**	**ACE**
Mean CAR Index	0.13	0.08	0.10	0.12	0.11	0.13	0.10
Max SST in Caribbean	0.36**	0.26*	0.22*	0.36**	0.31*	0.35**	0.27*
Min SST in Caribbean	0.72**	0.59**	0.47**	0.61**	0.55**	0.69**	0.56**
Max Latitude of 25°C	0.69**	0.56**	0.45**	0.59**	0.52**	0.66**	0.53**
Min Latitude of 25°C	0.72**	0.61**	0.44**	0.60**	0.58**	0.70**	0.58*
Max Latitude of 29°C	0.66**	0.52**	0.44**	0.60**	0.52**	0.62**	0.49**
Min Latitude of 29°C	-0.36**	-0.29*	-0.24*	-0.30*	-0.26*	-0.34*	-0.26*
Max SST at 24.5°N	0.69**	0.55**	0.44**	0.61**	0.53**	0.66**	0.52**
Max SST at 34.5°N	0.68**	0.53**	0.44**	0.61**	0.52**	0.65**	0.50**
Min SST at 34.5°N	0.75**	0.62**	0.48**	0.64**	0.58**	0.72**	0.58**

*p < 0.01 (compared to Pearson coefficient = 0). **p < 0.0001 (compared to Pearson coefficient = 0).

Table S2. Individual variable regression results, Wald χ^2 statistic.

	**0 month lag**	**3 month lag**	**6 month lag**	**12 month lag**	**18 month lag**	**24 month lag**
**Regional SST Variables**						
CAR Index	0.05	2.11	1.57	1.53	0.21	0.62
Max SST in Caribbean	0.23	0.01	2.86	2.37	1.94	3.06
Min SST in Caribbean	0	1.51	1.99	1.05	0.87	5.94*
**Warm SST Extent Variables**						
Max Latitude of 25 degrees	0.17	0.52	1.03	0.03	0.09	6.13*
Max Latitude of 29 degrees	3.24	0.85	0.29	4.05*	2.21	0.01
Min Latitude of 29 degrees	0.06	0.06	0.47	2.09	0.04	0.69
Min Latitude of 25 degrees	13.22*	11.34*	1.46	5.49*	3.44	5.9*
**SST at Parallel Variables**						
Max SST at 24.5N	1.84	0.02	1.64	0.89	0.27	0.46
Max SST at 34.5N	0.17	0.56	0.95	1.26	0.09	8.03*
Min SST at 24.5N	4.21	0.18	0.62	0.17	0.12	2.44
Min SST at 34.5N	0.6	2.42	6.97*	2.92	7.15*	6.86*
**Storm Variables**						
Total storms	0.4	0.01	0.58	0.15	4.22*	0.67
Hurricanes	1.21	0	0.01	0.61	0.8	0.1
Total storm-days	0.81	0.59	0.3	0.07	1.01	0.84
Tropical Depressions	2.08	1.27	2.27	0.29	0.62	3.04
Tropical Storms	1.52	0.12	0.07	0	2.34	0.05
Category 3 or greater storms	0.13	0.65	0.06	0.46	1.72	0.1
ACE	0.29	1.54	0.17	0.18	0.01	0.33

*$p < 0.05$ (compared to beta parameter = 0).

Table S2 displays the results of univariate logistic regression, evaluating the association between CFP calls and candidate explanatory storm and SST variables using 0, 3, 6, 12, 18, and 24 month time lag windows, controlling for month with dummy variables and using the yearly regional fishing yields as an offset. Wald chi-square statistics for the strength of association of the candidate weather variable with CFP calls are shown, with statistically significant associations indicated by asterisks. Beta parameters demonstrating the magnitude of association are shown in Table S3.

Table S3. Individual variable regression results, β estimate

	0 month lag	3 month lag	6 month lag	12 month lag	18 month lag	24 month lag
**Regional SST Variables**						
CAR Index	0.101	-0.655	-0.556	0.533	0.191	0.356
Max SST in Caribbean	0.107	0.024	-0.325	0.318	0.245	0.347
Min SST in Caribbean	-0.002	-0.115	-0.118	0.107	-0.078	-0.253*
**Warm SST Extent Variables**						
Max Latitude of 25 degrees	0.022	-0.034	-0.051	0.009	-0.015	-0.136*
Max Latitude of 29 degrees	0.067	-0.032	0.023	0.069*	0.058	0.004
Min Latitude of 29 degrees	-0.040	-0.028	0.109	-0.224	-0.029	-0.125
Min Latitude of 25 degrees	0.067*	0.062*	0.025	0.039*	0.039	0.042*
**SST at Parallel Variables**						
Max SST at 24.5N	0.222	-0.022	-0.201	0.162	0.087	0.124
Max SST at 34.5N	0.046	-0.076	-0.108	0.130	0.036	-0.336*
Min SST at 24.5N	0.251	-0.048	0.093	-0.047	0.040	0.192
Min SST at 34.5N	-0.099	-0.186	-0.291*	0.219	-0.310*	-0.345*
**Storm Variables**						
Total storms	-0.031	0.006	-0.046	0.019	0.112*	-0.042
Hurricanes	-0.084	-0.001	-0.007	0.057	0.081	-0.024
Total storm-days	-0.005	-0.006	-0.004	0.002	0.007	-0.006
Tropical Depressions	-0.227	0.205	-0.321	-0.075	0.132	-0.252
Tropical Storms	0.094	-0.035	-0.027	0.003	0.159	0.020
Category 3 or greater storms	-0.044	-0.132	-0.040	0.084	0.195	0.040
ACE	-0.002	-0.007	0.002	0.002	0.000	-0.002

*$p < 0.05$ (compared to beta parameter = 0).

Table S3 displays the results of univariate logistic regression, evaluating the association between CFP calls and candidate explanatory storm and SST variables using 0, 3, 6, 12, 18, and 24 month time lag windows, controlling for month with dummy variables and using the yearly regional fishing yields as an offset. Beta parameter estimates for the magnitude of association of the candidate weather variable with CFP calls are shown, with statistically significant associations indicated by asterisks. Wald chi-square statistics measuring the statistical strength of the association are shown in Table S2.

Table S4. Sensitivity of beta estimates to multivariate model selection.

**Dependent Variables Included in Model**	**Total Storms, 12 months lag Beta estimate (95% CI)**	**August Max SST, last year (5-16 month lag) Beta estimate (95% CI)**
**Final Model: Total storms (12 month lag), last year August Max SST, month dummies**	0.107 (0.003, 0.210)	0.481 (0.154, 0.808)
Final Model plus Min SST at 34.5N (12 month lag)	0.108 (0.009, 0.208)	0.677 (0.343, 1.011)
Final Model plus Min latitude of 25 degrees (12 month lag)	0.102 (-0.002, 0.205)	0.383 (0.013, 0.753)
Final Model plus March Min latitude of 25 degrees last year (10-21 month lag)	0.11 (0.005, 0.214)	0.396 (-0.065, 0.856)
Final Model plus Last March Min latitude of 25 degrees (1-12 month lag)	0.116 (0.014, 0.217)	0.392 (0.056, 0.727)
Final Model plus Max latitude of 29 degrees (12 month lag)	0.138 (0.024, 0.251)	0.433 (0.09, 0.777)
Final Model plus Min SST at 34.5N (12 month lag), Min latitude of 25 degrees (12 month lag)	0.103 (0.004, 0.203)	0.579 (0.205, 0.953)

Table S4 shows sensitivity analysis of the estimated effect of storms and SST in the final model to inclusion of additional candidate variables in the regression model. The beta estimates in the final model for storm and SST variables are shown in the first row with 95% CI, and beta estimates of the same variables when additional variables are included in the regression are shown in rows below.

Table S5. Sensitivity of rate ratios to outcome and offset restrictions.

Final model modified for sensitivity analysis	Total Storms, 12 months lag Rate Ratio (95% CI)	August Max SST, last year (5-16 month lag) Rate Ratio (95% CI)
Final model using only calls with moderate or severe clinical effect	1.03 (0.89, 1.19)	1.18 (0.76, 1.82)
Final model using 50 reef fish species fishing totals as offset	1.12 (1.02, 1.24)	1.37 (1.00, 1.88)
Final model using 13 reef fish species fishing totals as offset	1.13 (1.03, 1.24)	1.26 (0.94, 1.70)

Table S5 shows additional sensitivity analysis results. The first row shows rate ratios for final model storm and SST variables limiting outcome (CFP calls) to calls coded with only moderate or severe clinical effects (N = 412). The other models use limited fish species' yearly harvest data as offset for logistic regression instead of all fish. The model using 13 reef fish includes amberjacks, greater amberjacks, Atlantic Spanish mackerel, King mackerel, Crevalle jack, hogfish, Northern red snapper, Southern red snapper, porgies, red grouper, swordfish, tarpon, and barracuda. The model using 50 reef fish includes the above as well as multiple grouper species, multiple snapper species, multiple croaker species, multiple porgy species, parrotfishes, triggerfishes, seabass, additional jack species, surgeonfishes, additional mackerel species, multiple flounder species, and scamps.

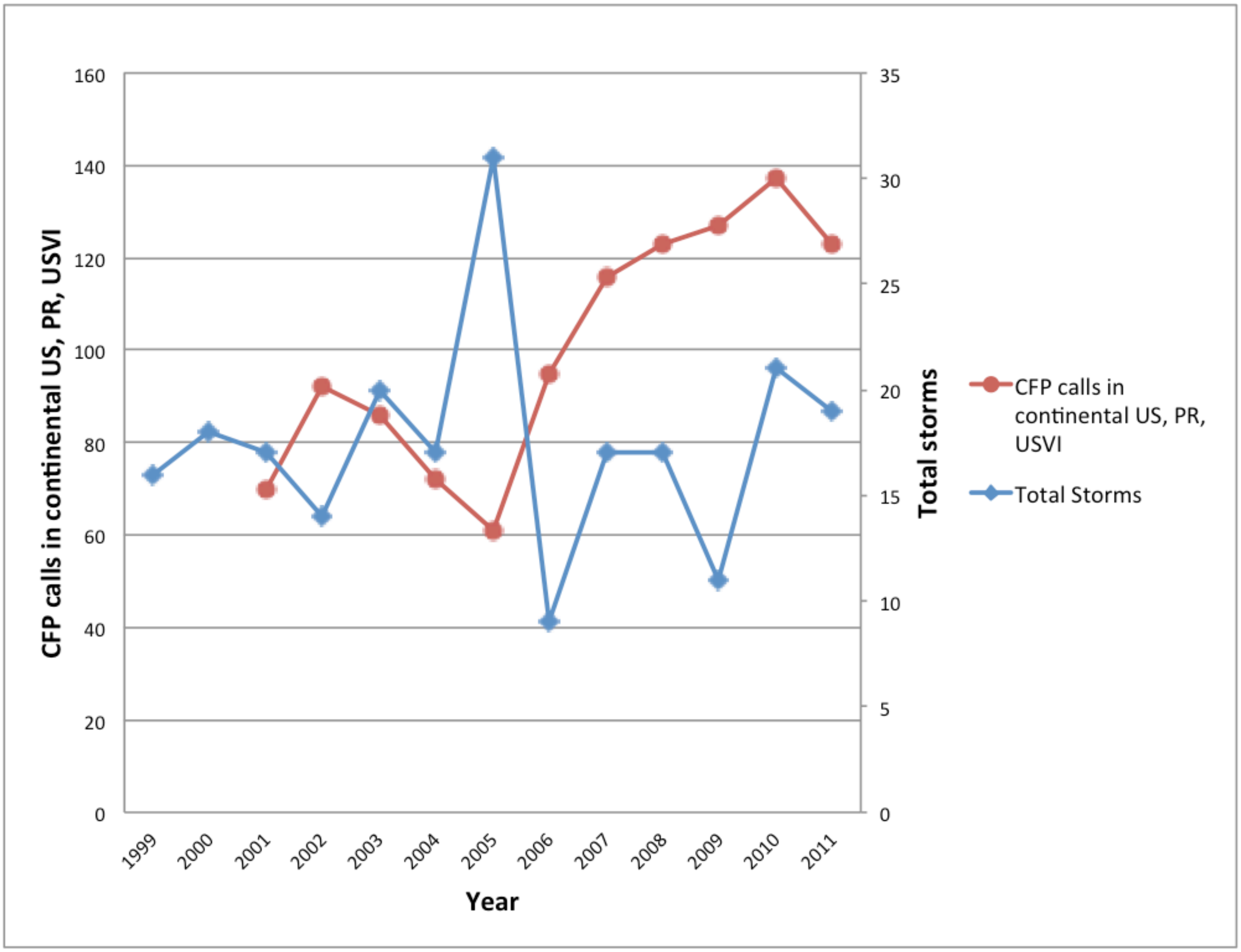

Figure S1. Total storms and CFP calls by year.

Figure S1 shows aggregate CFP calls to US poison control centers and total tropical storms time series years 1999-2011. CFP calls are only available from years 2001-2011, but weather data from years 1999-2000 were used to create lagged weather variables. Regression analysis was performed using monthly data with dummy variables controlling for month to compare across years. Total storms and August maximum SST are compared to calls in separate figures to simplify figure axes (see Figure S2).

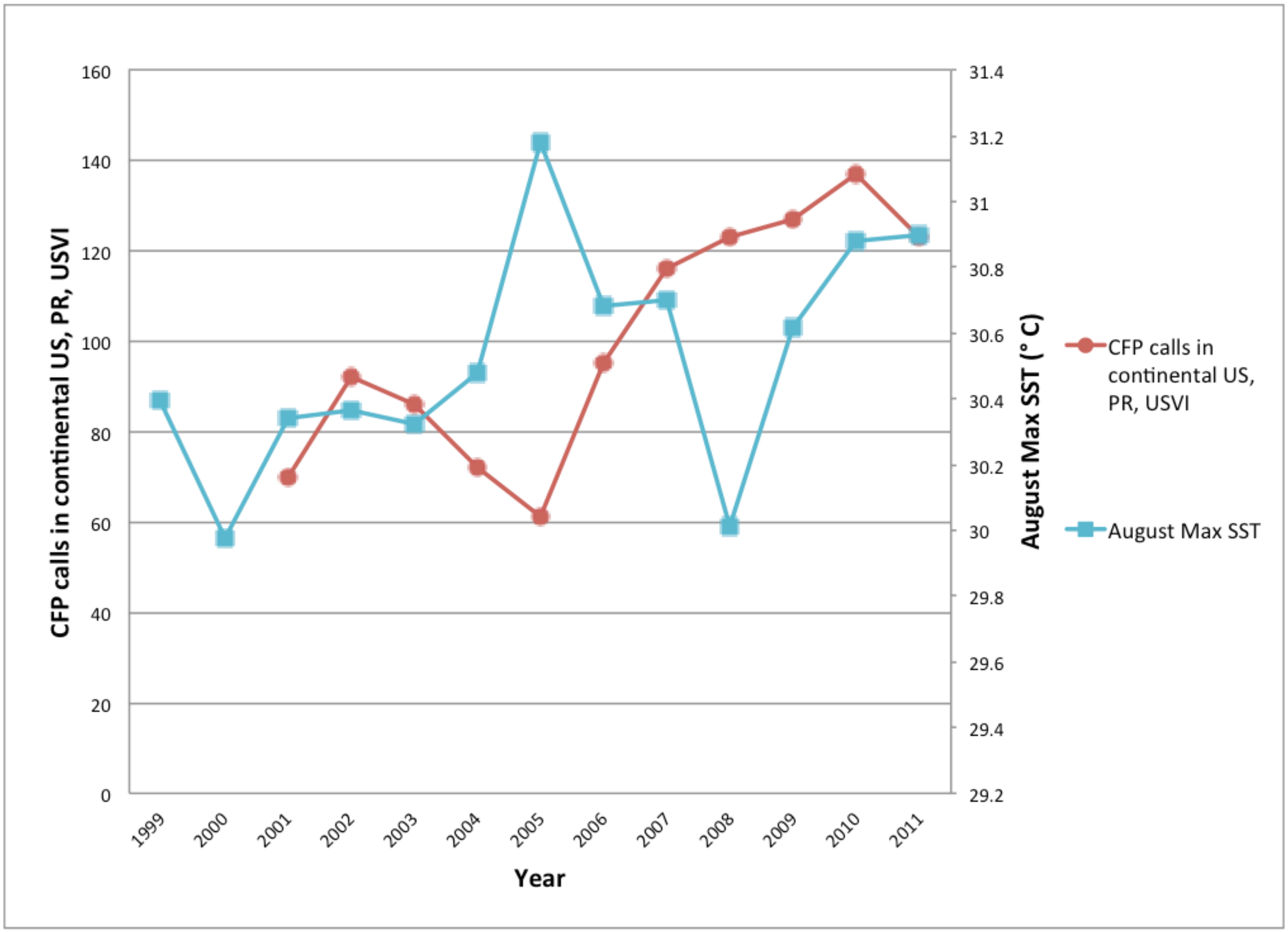

Figure S2. August max SST and CFP calls by year**.**

Figure S2 shows aggregate CFP calls to US poison control centers and the maximum SST in the Caribbean during August, years 1999-2000. CFP calls are only available from years 2001-2011, but weather data from years 1999-2000 were used to create lagged weather variables.
